# Detection of piroplasms infection in sheep, dogs and hedgehogs in Central China

**DOI:** 10.1186/2049-9957-3-18

**Published:** 2014-06-03

**Authors:** Zhuo Chen, Qin Liu, Feng-Chao Jiao, Bian-Li Xu, Xiao-Nong Zhou

**Affiliations:** 1 National Institute of Parasitic Diseases, Chinese Center for Disease Control and Prevention, Shanghai 200025, People’s Republic of China; 2 WHO Collaborative Center for Malaria, Schistosomiasis and Filariasis; Key Laboratory of Parasite and Vector Biology, Ministry of Health, Shanghai 200025, People’s Republic of China; 3 Xinyang College of Agriculture and Forestry, Xinyang 464000, People’s Republic of China; 4 Henan Center for Disease Control and Prevention, Zhengzhou 450016, People’s Republic of China

**Keywords:** *Babesia* spp, *Theileria* spp, Dogs, Sheep, Hedgehogs, China

## Abstract

**Background:**

Piroplasms are kinds of tick-borne parasitic apicomplexan protozoa, which are detrimental to humans and animals in tropical and subtropical areas around the world. Up until now, there has been a limited amount of reliable information available about the prevalence of piroplasms infections in wild animals in China. Therefore, we have investigated the infections of *Babesia* and *Theileria* species in both domestic and wild animals in Xinyang city, Henan province, where tick-borne diseases have recently been reported. This study aims to analyze the distribution patterns of piroplasms infections in animals, and assess their potential threat to humans in Central China.

**Methods:**

Blood samples were collected from sheep, dogs and hedgehogs in two regions, including Shihe District and Luoshan County, of Xinyang city, Henan province from August to December 2012. *Babesia* spp. and *Theileria* spp. were detected by polymerase chain reaction (PCR) and identified by sequencing and phylogenetic analysis. Moreover, the characteristics of detected piroplasms in different animal hosts were compared between the two study regions.

**Results:**

A total of 227 blood samples were collected from 73 sheep, two dogs and 152 hedgehogs. *Babesia* spp. was only detected in the two dogs. *Theileria* spp. was detected both in the sheep and the hedgehogs, and the total positive rate of *Theileria* spp. in the sheep and the hedgehogs was 57.53% and 13.82%, respectively. Sequencing and phylogenetic analysis revealed that the *Theileria* spp. detected in the sheep and the hedgehogs were very close to *T. lunwenshuni* cloned from a small ruminant and *Theileria* spp. isolated from a febrile hospitalized patient in China.

**Conclusion:**

*Babesia* and *Theileria* infections were detected in both domestic and wild animals in Xinyang city, Henan province in Central China, thus warranting further studies in these regions.

## Multilingual abstracts

Please see Additional file 1 for translations of the abstract into the six official working languages of the United Nations.

## Background

Piroplasmosis is a tick-borne disease which is caused by infection with apicomplexan protozoa, such as the *Babesia* or the *Theileria* species. These protozoan parasites are transmitted by vector ticks and can infect many different species of domestic and wild animals. Some species of the protozoan parasites are also pathogenic to humans [1-3].

Theileriosis and babesiosis are distributed widely in China. For instance, *B. caballi* and *T. equi* were detected in horses mainly in the northeastern and northwestern parts of China [4-6]. *T. buffeli, T. sergenti*, *B. bigemina, B. bovis, B. ovata* and *B. orientalis* were detected in cattle and buffalo in Central and southern China [7-9]. *T. ovis, T. uilenbergi, T. luwenshuni* and *B. motasi* were detected in sheep and goats mainly in Gansu, Ningxia, Qinghai, Henan, Inner Mongolia and Jilin provinces [10-13]. *B. gibsoni* and *B. canis vogeli* were detected in dogs in Jiangsu, Chongqing, Guangdong, Guangxi, Hainan and Zhejiang provinces [14,15]. *B. microti* infections were reported in animals and humans in the northeastern and southern parts of China [16,17]. However, only a limited amount of information is available on the prevalence of piroplasmosis in wild animals in China. There are only a few reports which documented small rodents infected with *B. microti* in Taiwan, Beijing, Zhejiang and Heilongjiang provinces [18-21] and Sika deer infected with *Theileria* spp. in Hubei province [22]. Some species of piroplasms may lead to high mortality and low productivity to susceptible animals, for instance, *T. annulata* was highly pathogenic to cattle [23]. Some other species, however, seemed to be benign to the host animals, and most of the infected animals did not show any clinical symptoms or signs of diseases [24].

*Babesia* spp., such as *B. microti* and *B. divergens*, are the major pathogenetic species of piroplasms causing human infections. However, some *Theileria* species can also cause severe acute diseases in humans as human theileriosis [25,26]. Previous research reported that 2.3% of 432 samples (from volunteers) were detected positive for *Theileria equi* antigens by indirect fluorescent antibody test (IFAT) [26]. Recently, a febrile hospitalized patient in Suizhou city of Hubei province was reported to be infected by a *Theileria* species (18S rRNA GenBank accession number HQ844673) by Liu et al. [27] which was very close to *T. lunwenshuni.*

Although piroplasmosis is one of the most prevalent diseases of domestic animals, the epidemiology and transmission characteristics among vector ticks and animal hosts are still unclear, and the situation would become even more complex if wild animals were acting as the natural reservoirs.

Thus, we investigated the infections of *Babesia* spp. and *Theileria* spp. in both domestic and wild animals in Xinyang city where tick borne diseases were recently reported, in order to analyze the distribution patterns of piroplasms infections in animals and assess their potential threat to humans in Central China.

## Methods

### Ethical clearance

The National Institute of Parasitic Diseases, Chinese Center for Disease Control and Prevention issued the ethical and institutional approval documents for this study.

### Sample collection

**Figure 1:**
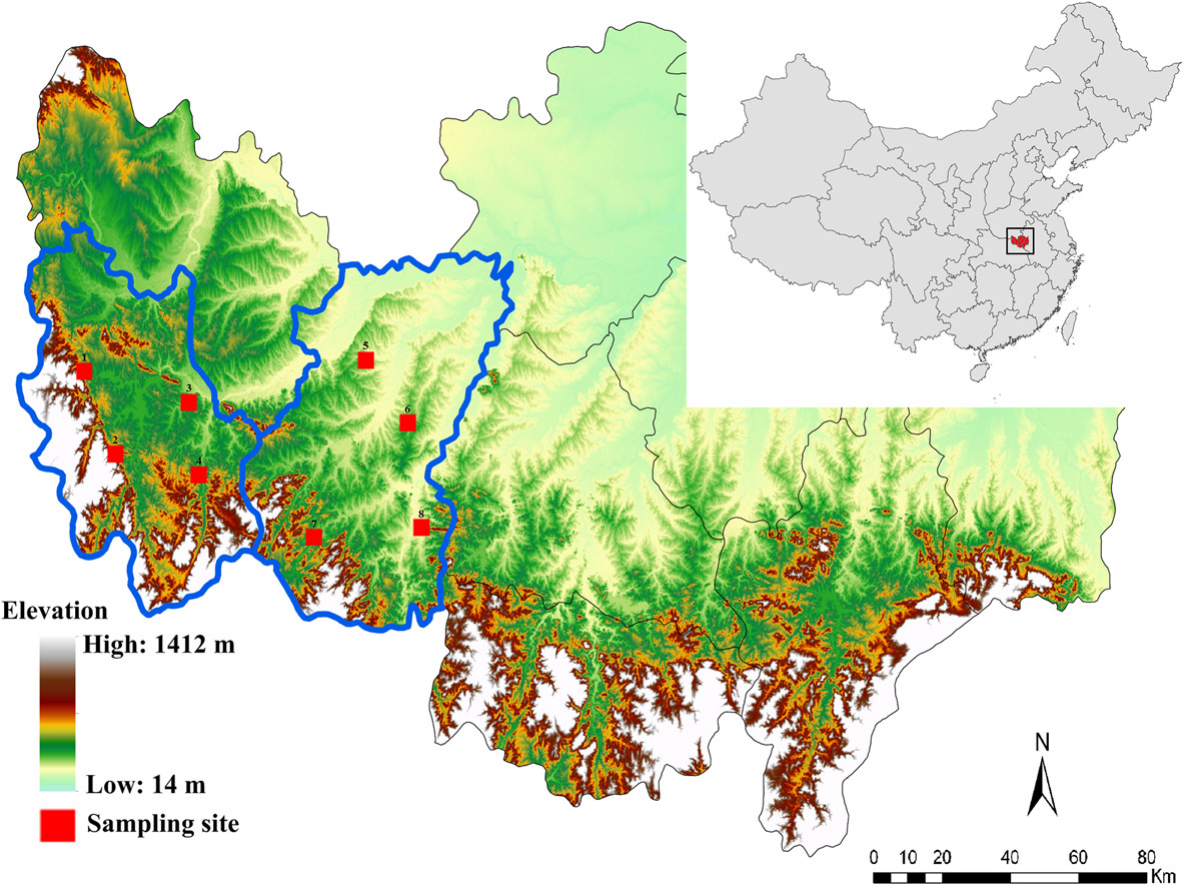
**The geographic map of sampling sites in Xinyang city of central China.** (Sampling sites 1–4 located in Shihe District and sampling sites 5–8 located in Luoshan County).

This study was carried out in Luoshan County and Shihe District of Xinyang city in Henan province from August to December 2012 (see Figure 1). The sampling sites were determined by a method of random grid sampling, which was performed using ArcGIS. A total of 227 blood samples were collected from 152 hedgehogs, 73 sheep and two dogs. Blood samples from the two dogs were collected from Shihe District. Detailed information on the numbers of blood samples collected from hedgehogs and sheep in each region is shown in Table 1. In this study, the veterinarians rescued the hedgehogs which were caught by the local people, and checked their health conditions. A very small amount of blood (less than 200 μl) was drawn from each animal, an amount that is harmless to the animal’s health. After this, the hedgehogs were released into their natural habitat. Blood samples were collected into an EDTA containing tube and kept frozen at −20°C until further processing.

**Table 1:** **Comparison of the differences in the positive rate of *Theileria *** **spp. in the sheep and the hedgehogs in the different locations**

**Location**	**Animal species**	**Number sampled**	**Number positive**	**Positive rate**	***χ***^**2**^	**DF**	**RR (95% CI)**	**P**
Shihe	Hedgehogs	51	19	37.25%	1.97	1	1.00	0.1600
	Sheep	53	27	50.94%			1.37 (0.88–2.13)	
Luoshan	Hedgehogs	101	2	1.98%	73.71	1	1.00	0.0000
	Sheep	20	15	75.00%			37.88 (9.38–152.87)	
Total	Hedgehogs	152	21	13.82%	46.75	1	1.00	0.0000
	Sheep	73	42	57.53%			4.16 (2.67–6.49)	

DF = degrees of freedom; RR = risk ratio; CI = confidence interval.

### DNA extraction and PCR amplification

Total genomic DNA was extracted from the whole blood of each sample using DNeasy Blood & Tissue Kit (Qiagen, Germany) according to the manufacturer’s instructions. Molecular detection of *Babesia* spp. and *Theileria* spp. was carried out using a nested PCR protocol targeting the 18S ribosomal RNA gene with specific primers in accordance with the method established by Silveira JA et al. [28]. Aliquot of double distilled water was used as the negative control to detect contamination. PCR was carried out in a C1000 Touch™ Thermal Cycler (BIO-RAD, USA). PCR products were separated by electrophoresis in 1.5% agarose gel. The positive PCR products were sent to Sangon Biotech (Shanghai, China) for sequencing in both directions.

### Phylogenetic analysis

The obtained 18S rRNA gene sequences were edited using the BioEdit 7.0.9 software. The pairwise nucleotide percent identity of the new sequences was calculated using MegAlign software (DNAStar Inc., Madison, WI, USA). The neighbor-joining tree was constructed using the MEGA 5.05 package [29]. Distances were estimated by the Kimura 2-parameter model and the numbers above the branch demonstrate bootstrap support from 1000 replications. The 18S rRNA gene sequence of *Cryptosporidium fragile* (EU1627541) was included in the tree as an outgroup.

### Statistical analysis

Differences in the positive rates of *Theileria* spp. in domestic and wild animals were tested by *χ*^2^-test, which was performed in SPSS 18.0.

## Results

### Detection of the Babesia spp. and the Theileria spp. infections in animals

Out of 227 samples, 63 (21 hedgehogs and 42 sheep) were detected positive for *Theileria* spp. in the two regions. *Babesia* spp. was only detected in the two dogs.

The positive rate of *Theileria* spp. in the hedgehogs and sheep in Shihe District were 37.25% and 50.94%, respectively (see Table 1). There was no statistically significant difference in the positive rate of *Theileria* spp. in the hedgehogs and sheep in Shihe District (*P*>0.05). However, there was statistically significant difference in the positive rate of *Theileria* spp. in the hedgehogs and sheep in Luoshan County. The positive rate of *Theileria* spp. in the hedgehogs and sheep in Luoshan County was 1.98% and 75.00%, respectively. The positive rate of *Theileria* spp. in the sheep was 37.88 times (*χ*^2^ = 73.71, DF = 1, *P* < 0.05) higher than that in the hedgehogs. The total positive rate of *Theileria* spp. in the hedgehogs and sheep in these two regions were 13.82% and 57.53%, respectively. The total positive rate of *Theileria* spp. in the sheep in these two regions was 4.16 times (*χ*^2^ = 46.75, DF = 1, *P* < 0.05) higher than that in the hedgehogs (see Table 1). There was no statistically significant difference in the positive rate of *Theileria* spp. in the sheep between the two regions. However, the positive rate of *Theileria* spp. in the hedgehogs in Shihe District was 18.81 times (*χ*^2^ = 35.41, DF = 1, *P* < 0.05) higher than that in Luoshan County (see Table 2).

**Table 2:** **Comparison of the differences in the positive rate of *Theileria *** **spp. in the sheep and hedgehog in Luoshan and Shihe**

**Animal species**	**Location**	**Number sampled**	**Number positive**	**Positive rate**	***χ***^**2**^	**DF**	**RR (95% CI)**	**P**
Sheep	Luoshan	20	15	75.00%	3.44	1	1.00	0.0636
	Shihe	53	27	50.94%			0.68 (0.47–0.98)	
Hedgehogs	Luoshan	101	2	1.98%	35.41	1	1.00	0.0000
	Shihe	51	19	37.25%			18.81 (4.56–77.65)	

DF = degrees of freedom; RR = risk ratio; CI = confidence interval.

### Sequencing and phylogenetic analysis

**Figure 2:**
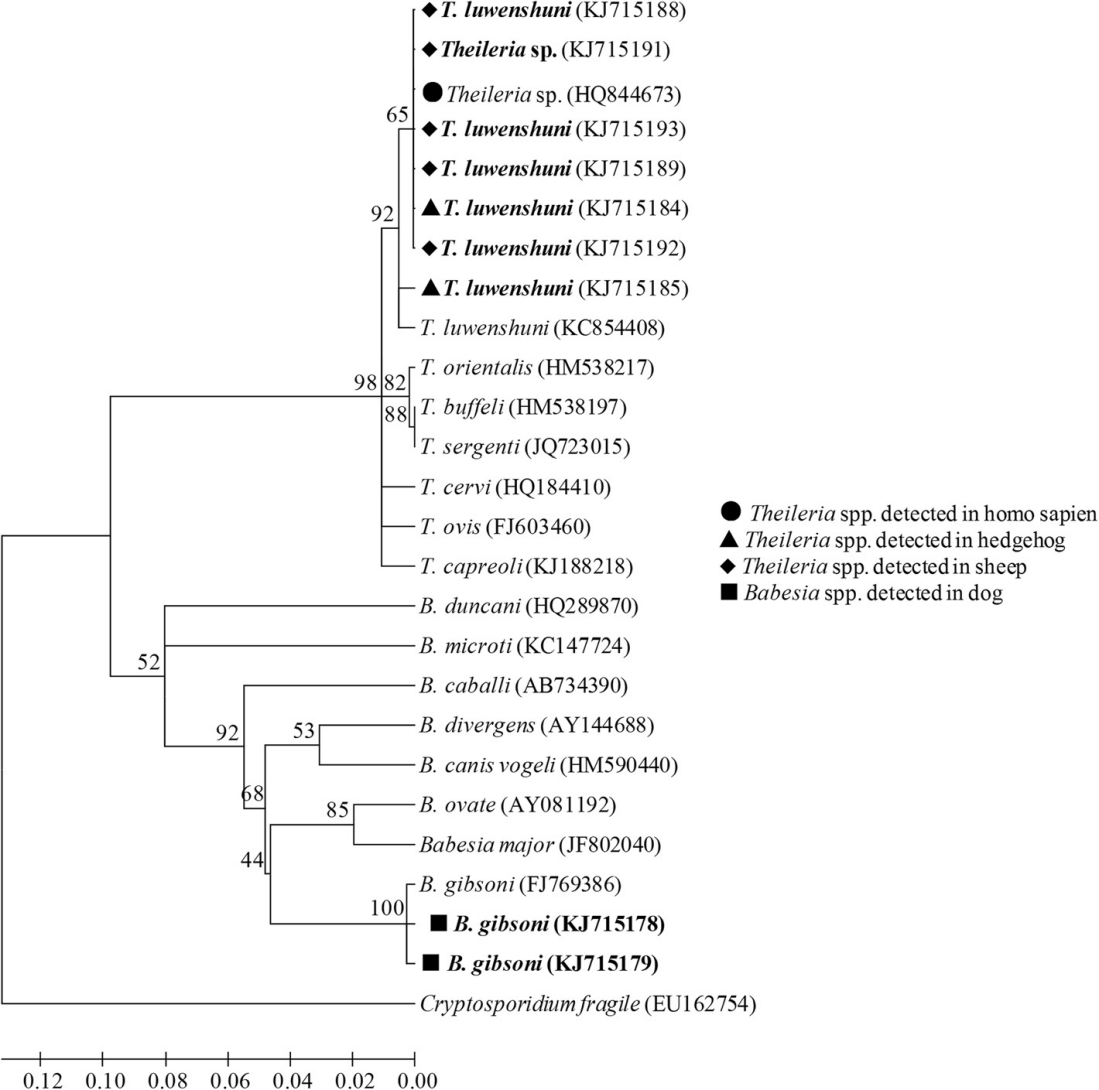
**Neighbor-joining tree showing the phylogenetic relationship of the 18S rRNA gene sequences of *Theileria *and *Babesia *species identified in this study and those present in the GenBank database.** (The GenBank accession numbers are indicated in parentheses. Species detected in this study are indicated in bold. The scale bar represents nucleotide substitutions per position).

*T. luwenshuni* was identified in 61 samples, 15 sheep and two hedgehogs in Luoshan County, and 26 sheep and 18 hedgehogs in Shihe District. *Theileria* sp. was detected in one hedgehog and one sheep respectively in Shihe District. *B. gibsoni* was identified in the two dogs in Shihe District. The sequences of detected piroplasms in this study were deposited into GenBank. The accession numbers are KJ715184, KJ715185, KJ715188, KJ715189, KJ715191, KJ715192, KJ715193, KJ715178 and KJ715179 (see Figure 2).

The obtained sequences were edited and assembled to a final length of 350 base pairs (bp). All the *Theileria* spp. detected in this study had 94.6%-99.7% identity with nucleotide differences of 1–19 bp (see Table 3). The constructed phylogenetic tree of maximum-likelihood analysis depicting the relationships of the 18S rRNA gene of the *Theileria* and *Babesia* species identified in this study and those present in the GenBank database is shown in Figure 2. The sequences of *Theileria* spp. in this study fall into the same clade with *T. lunwenshuni* (KC854408) cloned from a small ruminant in China and *Theileria* sp. (HQ844673) isolated from a febrile hospitalized patient in China (see Figure 2). The sequences of *Babesia* spp. are in the same clade with *B. gibsoni* (FJ769386) cloned from a dog in Taiwan.

**Table 3:** **Nucleotide percent identity between the 18S rRNA of *Theileria *** **spp** ***. *** **detected in this study**

**Sequence**		**1**	**2**	**3**	**4**	**5**	**6**
1	KJ715184						
2	KJ715185	96.3					
3	KJ715188	98.9	96.3				
4	KJ715189	98.9	96.0	98.9			
5	KJ715191	98.6	95.4	98.9	98.3		
6	KJ715192	98.6	94.6	98.6	98.9	98.3	
7	KJ715193	98.9	96.3	99.7	98.9	99.1	99.1

## Discussion

In this study, we found that 27.11% (61/225) samples from sheep and hedgehogs were positive for *T. luwenshuni*. This result suggests that *T. luwenshuni* was the predominant *Theileria* species in both hedgehogs and sheep in the two studied regions. This study is the first to report on the identification of *T. luwenshuni* in small wild mammals in Central China. Other *Theileria* species, e.g. *T. uilenbergi* and *T. ovis*, have been reported in the southeastern parts of China [30], but were not found in this study. This may possibly be associated with the ecosystem features of the local environment.

It was reported that *T. lunwenshuni* was transmitted by *Haemaphysalis qinghaiensis*[31], and small ruminant animals were infected with it in the northern and central parts of China [32], with high pathogenicity causing great economic losses. Animals that survived acute infection could become low-level carriers of the parasite, and would remain persistently infected for years without apparent clinical signs [33]. In the present study, all the animals positive for *T. lunwenshuni* did not show any clinical symptoms or signs of the disease. However, if vector ticks feed on these sub-clinical carriers, they can be infected and transmit the pathogen to exotic animals [33], and even to human beings.

One exciting finding of this study was, as revealed by the phylogenetic analysis, that the *Theileria* spp. detected in this study was very close to the *Theileria* sp. isolated from a febrile hospitalized patient in Suizhou city of Hubei province. In addition, Suizhou city is adjacent to Shihe District geographically. Thus, whether the pathogens were different strains of the same species, whether they would be pathogenic to different species of host animals, or whether they could be a potential threat to human beings is still unclear, and this finding warrants further studies.

In this epidemiological study on piroplasms infection, it is important to understand the role of the different host animals in the transmission cycle [34-36]. We found that the total positive rate of *Theileria* spp. in the sheep in the two study regions was much higher than that in the hedgehogs, indicating that domestic animals might play a more essential role in the transmission of piroplasmosis. This result was supported by the following facts: First, the population density of domestic animals is much higher than that of wild animals. In fact, sheep husbandry is very common in the rural areas of the study regions. A total of 818.4 thousands of sheep were raised in Xinyang city, based on the data from the Henan Statistical Yearbook 2012. These huge numbers of sheep posed a high risk of exposure and infection to livestock, and increase the economic burden on animal husbandry and farmers in these regions. Second, the habits of animals are indicative of their exposures to infections. Although the population size of hedgehogs in these regions is unavailable, the living habits of hedgehogs are definitely different from domestic animals as hedgehogs are not gregarious animals [37-40]. This may reduce the risk of exposure to tick vectors. Moreover, the positive rate of *Theileria* spp. in the hedgehogs in Shihe District was much higher than that in Luoshan County, it also indicated that the population density of sheep in Shihe District might be much higher than that in Luoshan County. Sheep might live in closer contact with hedgehogs in Shihe District due to the increased environmental and social economic activities.

In this study, *Babesia* spp. was only detected in dogs*.* This could be attributed to only one transmission season, as well as to the limited number of host species that was taken into account. However, babesiosis had been reported in livestock in Henan province [41]. Previous studies on ticks [42] and rodents [43] collected in Xinyang city documented the detection of *B. microti*, which could cause babesiosis in animals and humans. Therefore, it is necessary to carry out more studies on the epidemiology of *Babesia* spp. and *Theileria* spp. infections in more species of domestic and wild animals, as well as on the infection status of vector ticks in these regions.

## Conclusion

*Babesia* spp. and *Theileria* spp. infections were detected in both domestic and wild animals in the study regions of Central China. Further studies are needed to estimate the impacts to local animal husbandry by piroplasms infection and to establish biological measures to control the vector ticks.

## Competing interests

The authors declare that they have no competing interests.

## Authors’ contributions

ZC conducted the field sampling, performed the laboratory work, generated experimental data, and wrote the manuscript. QL, X-NZ and B-LX had a substantial role in the conception of the study, guidance of the practical work and writing of the manuscript. F-CJ helped with the sample collection. All authors read and approved the final manuscript.

## Supplementary Material

Additional file 1 Multilingual abstracts in the six official working languages of the United Nations. Click here for file
