# Supplementary material for: Detection of piroplasms infection in sheep, dogs and hedgehogs in Central China
Source: Infect Dis Poverty. 2014 Jun 3;3:18. doi: 10.1186/2049-9957-3-18 (PMC4051148; doi:10.1186/2049-9957-3-18)
Supplement: Additional file 1 — Multilingual abstracts in the six official working languages of the United Nations. [file 2049-9957-3-18-S1.pdf]

Translation of the abstract into the six official working languages of the United Nations

اكتشاف إصابة الأغنام والكلاب والقنافذ بمرض البيروبلاسما في وسط الصين

زيو شن، كن ليو، فنغ شياو جياو، بيان لي كسو، كسياو نونغ زو

ملخص
معلومات أساسية: إن البيروبلاسما هو مرض طفيلي تنقله القراد وهي طلائعية أبيكومبلاكسن والتي تضر بالإنسان والحيوان في المناطق الاستوائية وشبه الاستوائية حول العالم. إلى حد اليوم، فإن المعلومات المتعلقة بانتشار عدوى بيروبلاسما بين الحيوانات البرية في الصين الموثوق بها والمنشورة محدودة. لذلك، فقد قمنا بالتحقيق في أنواع الالتهابات البابسية والتيلرية لدى الحيوانات الأليفة والبرية في مدينة اكسينيانج بمقاطعة خنان أين تم الإبلاغ مؤخرا عن أمراض ينقلها القراد.
تهدف هذه الدراسة إلى تحليل أنماط توزيع الإصابات بمرض البيروبلاسما لدى الحيوانات وتقييم التهديد المحتمل للإنسان في وسط الصين.
الأساليب: تم جمع عينات من دم الأغنام والكلاب والقنافذ في منطقتين بما في ذلك منطقة شيخه ومقاطعة لوشان ومدينة اكسينيانج بمقاطعة خنان وذلك من شهر أوت(أغسطس) إلى شهرديسمبر(كانون الأول) لسنة 2012. وقد تم اكتشاف الإلتهابات البابسية والتيلرية عن طريق تفاعل البوليميراز المتسلسل(PCR) وتم تحديدها حسب تحليل النشوء والتطور.علاوة على ذلك، تمت المقارنة بين خصائص البيروبلاسما التي تم اكتشافها لدى حيوانات مختلفة حاملة لهذا المرض في المنطقتين محل الدراسة.
النتائج: لقد تم اكتشاف العدوى البابسية و التيلرية في كل من الحيوانات الأليفة والبرية من مدينة شينغيانغ بمقاطعة خنان في وسط الصين مما يبرر القيام بمزيد من الدراسات في المنطقة.

Translated from English version into Arabic by malika2012, through

TRANSLATORS WITHOUT BORDERS

中国中部地区羊、狗和刺猬中梨形虫感染调查

陈卓，刘琴，焦凤超，许汴利，周晓农

摘要

引言：梨形虫是经蜱传播的顶复门原虫，广泛分布于热带和亚热带地区，对人畜均有较大危害。目前，我国野生动物中梨形虫感染的研究报道相对较少。因此，我们调查了河南省信阳地区家畜和野生动物中巴贝虫和泰勒虫感染情况。旨在了解动物感染梨形虫的分布情况并评估其对当地居民和家畜的影响。

方法：2012 年 8-12 月间，在河南省信阳市的浉河区和罗山县采集羊，狗和刺猬的血液样本。采用巢式 PCR，DNA 测序和系统发育分析的方法检测巴贝虫和泰勒虫感染，并对不同地区不同动物宿主中的梨形虫感染情况进行比较分析。

结果：共采集到 227 份血液样本，包括 73 只羊，2 只狗和 152 只刺猬。经检测两只狗均只感染了巴贝虫，感染率为 100%。羊和刺猬中只检测到泰勒虫感染，总感染率分别为 57.53%和 13.82%。经测序和系统进化分析，信阳地区羊和刺猬中检测到的泰勒虫与从湖北随州市反刍动物中及当地一例发热病人中分离到的泰勒虫相似，且均与吕氏泰勒虫高度同源。

结论：信阳地区的家畜和野生动物中检测到巴贝虫和泰勒虫感染，其对当地居民和动物的影响还有待进一步研究。

Translated from English version into Chinese by Chen Zhuo

Détection d'infections de piroplasmes chez les moutons, les chiens et les hérissons dans le centre de la Chine

Zhuo Chen, Qin Liu, Feng-Chao Jiao, Bian-Li Xu, Xiao-Nong Zhou

Résumé

Contexte: Les piroplasmes sont des types de protozoaires parasites apicomplexes transmis par les tiques pouvant nuire aux humains et aux animaux dans les régions tropicales et subtropicales. Jusqu'à présent, il y a peu d'informations fiables disponibles sur la prévalence des infections de piroplasmes chez les animaux sauvages en Chine. Nous avons donc étudié les espèces de *Babesia* et de *Theileria* chez les animaux domestiques et sauvages de la ville de Xinyang de la province du Henan où des maladies transmises par les tiques ont récemment été signalées. Cette étude vise à analyser les profils de répartition des cas d'infections de piroplasmes chez les animaux et à évaluer une menace potentielle pour les humains dans le centre de la Chine.

Méthodes: Des échantillons de sang ont été prélevés chez les moutons, les chiens et les hérissons d'août à décembre 2012 dans deux régions, dont le district de Shihe et le comté de Luoshan de la ville de Xinyang dans la province du Henan. Le *Babesia* spp. et le *Theileria* spp. ont été détectés par une réaction en chaîne de la polymérase (RCP) et identifiés par le séquençage et l'analyse phylogénétique. De plus, les caractéristiques des piroplasmes détectés dans les différents hôtes animaux ont été comparées entre les deux régions à l'étude.

Résultats: Au total, 227 échantillons de sang ont été prélevés de 73 moutons, 2 chiens et 152 hérissons. Le *Babesia* spp. a uniquement été détecté chez les deux chiens. Le *Theileria* spp. a été détecté chez les moutons et les hérissons, et le taux positif total de *Theileria* spp. chez les moutons et les hérissons étaient de 57,53 % et de 13,82 % respectivement. Le séquençage et l'analyse phylogénétique ont révélé que le *Theileria* spp. détecté dans les moutons et les hérissons dans le cadre de cette étude était très prêt du *T. lunwenshuni* cloné à partir de petits ruminants et du *Theileria* sp. isolé à partir d'un patient fiévreux hospitalisé en Chine.

Conclusion: Des infections de *Babesia* et de *Theileria* ont été détectées chez les animaux domestiques et sauvages de la ville de Xingyang de la province du Henan dans le centre de la Chine, justifiant ainsi des études plus approfondies dans la région.

Translated from English version into French by Melissa Guay, through

TRANSLATORS
WITHOUT BORDERS

Обнаружение инфицирования пироплазмами у овец, собак и ежей в Центральном Китае

Чжо Чен, Цинь Лу, Фэн-Чжао Цзяо, Бянь-Ли Сюй, Сяо-Нун Чжу

Краткое изложение

История вопроса: Пироплазмы – это виды передаваемых клещами паразитарных простейших одноклеточных организмов, несущих угрозу здоровью человека и животных в тропических и субтропических зонах по всему миру. На настоящий момент в Китае существует очень ограниченное количество достоверной информации о распространении заражения пироплазмами среди диких животных. Поэтому мы исследовали инфицирование видами *Babesia* и *Theileria* среди домашних и диких животных в городе Синьян, провинции Хэнань, где недавно наблюдались передаваемые клещами заболевания. Данное исследование анализирует схемы распространения инфицирования пироплазмами животных и оценивает потенциальную угрозу человеку в Центральном Китае.

Методы: Образцы крови были забраны у овец, собак и ежей в двух районах, район Шихэ и уезд Лошань, в городе Синьян, провинции Хэнань, в период с августа по декабрь 2012 г. *Babesia* spp. и *Theileria* spp. были обнаружены способом полимеразной цепной реакции (ПЦР) и опознаны путем секвенирования и филогенетического анализа. Кроме этого, было проведено сравнение обнаруженных пироплазмов в различных животных-носителях между двумя рассматриваемыми районами.

Результаты: Было забрано 227 образцов крови у 73 овец, 2 собак и 153 ежей. *Babesia* spp. были обнаружены только у двух собак. *Theileria* spp. были обнаружены у овец и ежей, и общий положительный показатель инфицирования *Theileria* spp. в овцах и ежах составил 57,53% и 13,82% соответственно. Секвенирование и филогенетический анализ показали, что *Theileria s*pp., обнаруженные в данном исследовании у овец и ежей, были очень близки к *T. lunwenshuni*, клонированным с мелких жвачных животных, и *Theileria* sp., полученных с лихорадочного госпитализированного пациента в Китае.

Заключение: Инфицирование видами *Babesia* и *Theileria* было обнаружено как в домашних, так и в диких животных в городе Синьян, провинции Хэнань в Центральном Китае, что подтвердило необходимость дополнительных

исследований в данном регионе.

Translated from English version into Russian by Elena McDonnell, through

TRANSLATORS
WITHOUT BORDERS

Detección de infección por piroplasmas en ovejas, perros y erizos en la región central de China

Zhuo Chen, Qin Liu, Feng-Chao Jiao, Bian-Li Xu, Xiao-Nong Zhou

Resumen

Antecedentes: Los piroplasmas son tipos de protozoos parásitos apicomplexa transmitidos por garrapatas que son perjudiciales para humanos y animales en regiones tropicales y subtropicales en todo el mundo. Hasta el momento, existe información confiable limitada, disponible en China, sobre la preponderancia de infecciones por piroplasmas en animales salvajes. Por lo tanto, investigamos infecciones de especies de *Babesia* y *Theileria* tanto en animales domésticos como en los salvajes en la ciudad de Xinyang, provincia de Henan donde recientemente se han registrado enfermedades transmitidas por garrapatas. El objetivo de este estudio es analizar los patrones de distribución de las infecciones por piroplasmas en animales y evaluar la amenaza potencial a humanos en la región central de la China.

Métodos: Se recogieron muestras de sangre de ovejas, perros y erizos en dos regiones, incluyendo el distrito Shihe y el condado de Luoshan, de la ciudad de Xinyang, provincia de Henan desde agosto hasta diciembre de 2012. Se detectó *Babesia* spp. y *Theileria* spp. por la reacción en cadena de polimerasas (PCR, por sus siglas en inglés) y se identificaron por análisis secuencial y filogenético. Por otra parte, las características de los piroplasmas detectados en huéspedes de animales diferentes fueron comparadas entre las dos regiones bajo estudio.

Resultados: Fueron recogidas un total de 227 muestras de sangre de 73 ovejas, 2 perros y 152 erizos. *Babesia* spp. fue detectada solamente en dos perros. *Theileria* spp. fue detectada en ovejas y erizos y el índice positivo total de *Theileria* spp. en ovejas y erizos fue de 57,53% y 13,82%, respectivamente. Los análisis secuenciales y filogenéticos revelaron que la *Theileria s*pp. detectada en ovejas y erizos en este estudio fueron muy similares a los de *T. lunwenshuni* clonados de rumiantes pequeños y *Theileria* sp. aislados a partir de un paciente febril hospitalizado en China.

Conclusiones: Las infecciones por *Babesia* y *Theileria* fueron detectadas tanto en animales domésticos como en los salvajes de la ciudad de Xingyang, provincia de Henan en la región central de China, lo que garantizó estudios adicionales en la región.

Translated from English version into Spanish by two2tango, through

TRANSLATORS
WITHOUT BORDERS
